# Supplementary material for: Effect of floods on the δ13C values in plant leaves: a study of willows in Northeastern Siberia
Source: PeerJ. 2018 Sep 20;6:e5374. doi: 10.7717/peerj.5374 (PMC6151259; doi:10.7717/peerj.5374)
Supplement: Supplemental Information 1 [file peerj-06-5374-s001.pptx]

## Slide 1
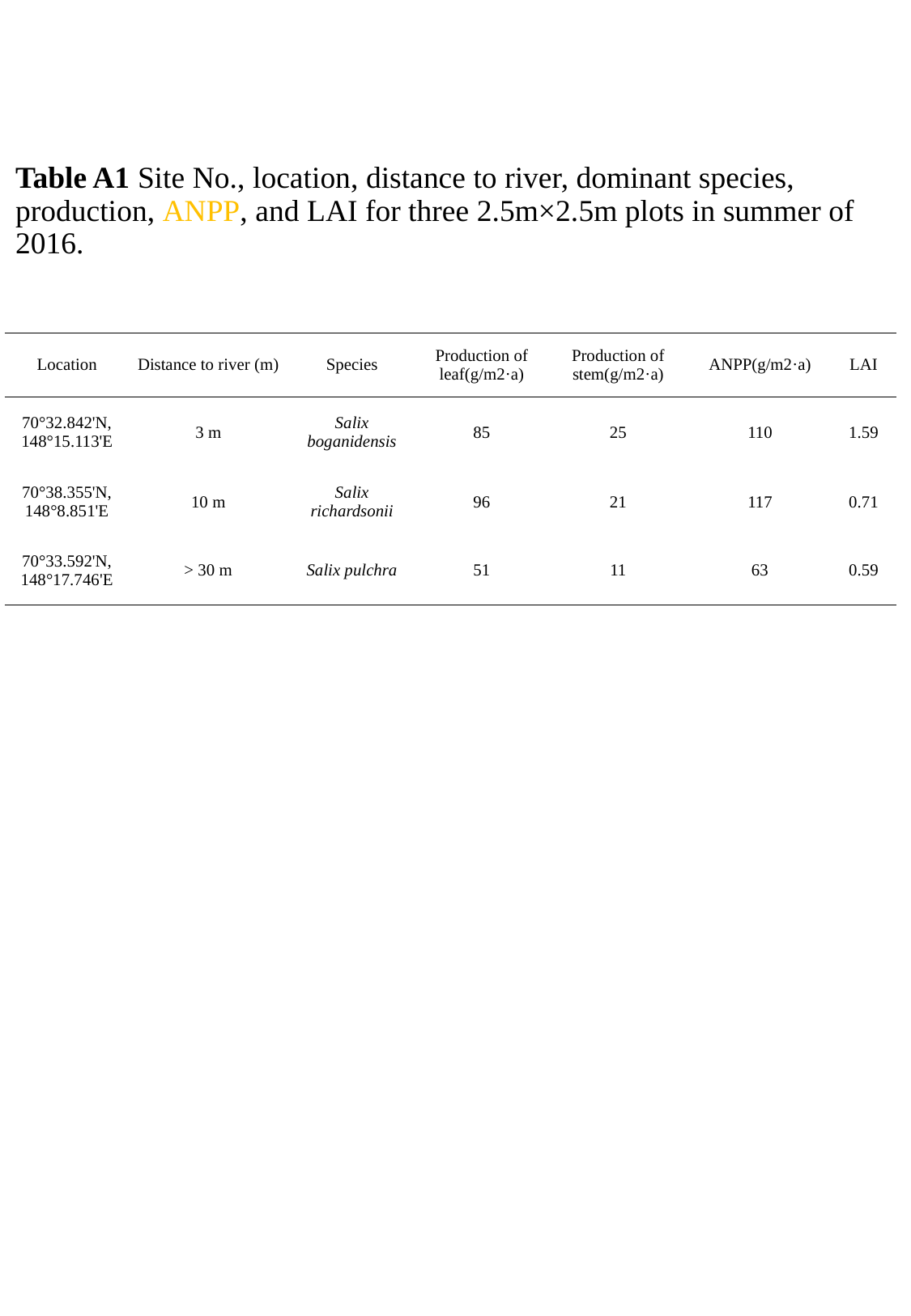

# Table A1 Site No., location, distance to river, dominant species, production, ANPP, and LAI for three 2.5m×2.5m plots in summer of 2016.
| Location | Distance to river (m) | Species | Production of leaf(g/m2·a) | Production of stem(g/m2·a) | ANPP(g/m2·a) | LAI |
| --- | --- | --- | --- | --- | --- | --- |
| 70°32.842'N, 148°15.113'E | 3 m | Salix boganidensis | 85 | 25 | 110 | 1.59 |
| 70°38.355'N, 148°8.851'E | 10 m | Salix richardsonii | 96 | 21 | 117 | 0.71 |
| 70°33.592'N, 148°17.746'E | > 30 m | Salix pulchra | 51 | 11 | 63 | 0.59 |
