# Supplementary material for: Effect of floods on the δ13C values in plant leaves: a study of willows in Northeastern Siberia
Source: PeerJ. 2018 Sep 20;6:e5374. doi: 10.7717/peerj.5374 (PMC6151259; doi:10.7717/peerj.5374)
Supplement: Supplemental Information 2 — I and K river represent Indigirka and Kryvaya river. [file peerj-06-5374-s002.pptx]

## Slide 1
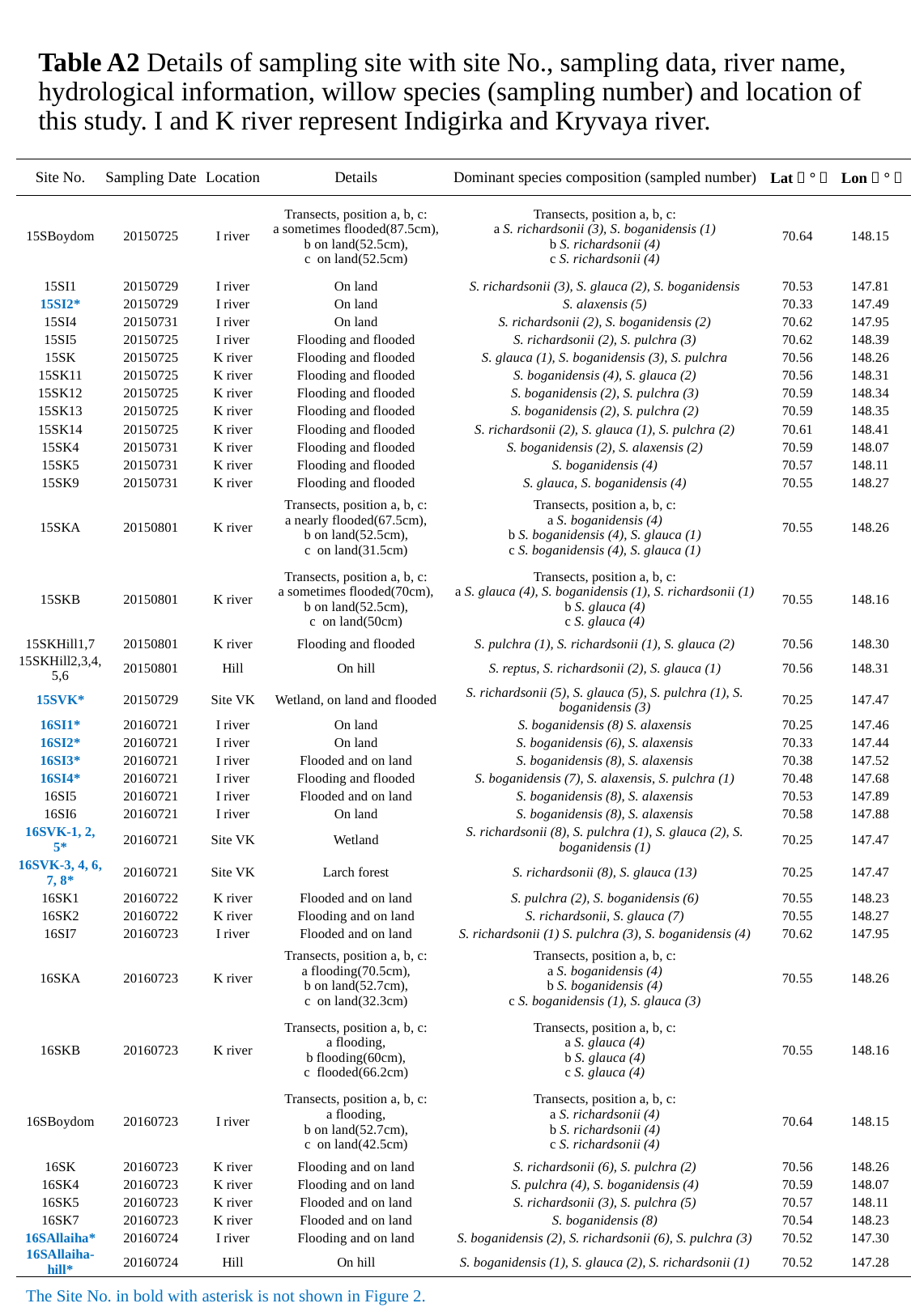

# Table A2 Details of sampling site with site No., sampling data, river name, hydrological information, willow species (sampling number) and location of this study. I and K river represent Indigirka and Kryvaya river.
| Site No. | Sampling Date | Location | Details | Dominant species composition (sampled number) | Lat（°） | Lon（°） |
| --- | --- | --- | --- | --- | --- | --- |
| 15SBoydom | 20150725 | I river | Transects, position a, b, c: a sometimes flooded(87.5cm), b on land(52.5cm), c on land(52.5cm) | Transects, position a, b, c: a S. richardsonii (3), S. boganidensis (1) b S. richardsonii (4) c S. richardsonii (4) | 70.64 | 148.15 |
| 15SI1 | 20150729 | I river | On land | S. richardsonii (3), S. glauca (2), S. boganidensis | 70.53 | 147.81 |
| 15SI2\* | 20150729 | I river | On land | S. alaxensis (5) | 70.33 | 147.49 |
| 15SI4 | 20150731 | I river | On land | S. richardsonii (2), S. boganidensis (2) | 70.62 | 147.95 |
| 15SI5 | 20150725 | I river | Flooding and flooded | S. richardsonii (2), S. pulchra (3) | 70.62 | 148.39 |
| 15SK | 20150725 | K river | Flooding and flooded | S. glauca (1), S. boganidensis (3), S. pulchra | 70.56 | 148.26 |
| 15SK11 | 20150725 | K river | Flooding and flooded | S. boganidensis (4), S. glauca (2) | 70.56 | 148.31 |
| 15SK12 | 20150725 | K river | Flooding and flooded | S. boganidensis (2), S. pulchra (3) | 70.59 | 148.34 |
| 15SK13 | 20150725 | K river | Flooding and flooded | S. boganidensis (2), S. pulchra (2) | 70.59 | 148.35 |
| 15SK14 | 20150725 | K river | Flooding and flooded | S. richardsonii (2), S. glauca (1), S. pulchra (2) | 70.61 | 148.41 |
| 15SK4 | 20150731 | K river | Flooding and flooded | S. boganidensis (2), S. alaxensis (2) | 70.59 | 148.07 |
| 15SK5 | 20150731 | K river | Flooding and flooded | S. boganidensis (4) | 70.57 | 148.11 |
| 15SK9 | 20150731 | K river | Flooding and flooded | S. glauca, S. boganidensis (4) | 70.55 | 148.27 |
| 15SKA | 20150801 | K river | Transects, position a, b, c: a nearly flooded(67.5cm), b on land(52.5cm), c on land(31.5cm) | Transects, position a, b, c: a S. boganidensis (4) b S. boganidensis (4), S. glauca (1) c S. boganidensis (4), S. glauca (1) | 70.55 | 148.26 |
| 15SKB | 20150801 | K river | Transects, position a, b, c: a sometimes flooded(70cm), b on land(52.5cm), c on land(50cm) | Transects, position a, b, c: a S. glauca (4), S. boganidensis (1), S. richardsonii (1) b S. glauca (4) c S. glauca (4) | 70.55 | 148.16 |
| 15SKHill1,7 | 20150801 | K river | Flooding and flooded | S. pulchra (1), S. richardsonii (1), S. glauca (2) | 70.56 | 148.30 |
| 15SKHill2,3,4,5,6 | 20150801 | Hill | On hill | S. reptus, S. richardsonii (2), S. glauca (1) | 70.56 | 148.31 |
| 15SVK\* | 20150729 | Site VK | Wetland, on land and flooded | S. richardsonii (5), S. glauca (5), S. pulchra (1), S. boganidensis (3) | 70.25 | 147.47 |
| 16SI1\* | 20160721 | I river | On land | S. boganidensis (8) S. alaxensis | 70.25 | 147.46 |
| 16SI2\* | 20160721 | I river | On land | S. boganidensis (6), S. alaxensis | 70.33 | 147.44 |
| 16SI3\* | 20160721 | I river | Flooded and on land | S. boganidensis (8), S. alaxensis | 70.38 | 147.52 |
| 16SI4\* | 20160721 | I river | Flooding and flooded | S. boganidensis (7), S. alaxensis, S. pulchra (1) | 70.48 | 147.68 |
| 16SI5 | 20160721 | I river | Flooded and on land | S. boganidensis (8), S. alaxensis | 70.53 | 147.89 |
| 16SI6 | 20160721 | I river | On land | S. boganidensis (8), S. alaxensis | 70.58 | 147.88 |
| 16SVK-1, 2, 5\* | 20160721 | Site VK | Wetland | S. richardsonii (8), S. pulchra (1), S. glauca (2), S. boganidensis (1) | 70.25 | 147.47 |
| 16SVK-3, 4, 6, 7, 8\* | 20160721 | Site VK | Larch forest | S. richardsonii (8), S. glauca (13) | 70.25 | 147.47 |
| 16SK1 | 20160722 | K river | Flooded and on land | S. pulchra (2), S. boganidensis (6) | 70.55 | 148.23 |
| 16SK2 | 20160722 | K river | Flooding and on land | S. richardsonii, S. glauca (7) | 70.55 | 148.27 |
| 16SI7 | 20160723 | I river | Flooded and on land | S. richardsonii (1) S. pulchra (3), S. boganidensis (4) | 70.62 | 147.95 |
| 16SKA | 20160723 | K river | Transects, position a, b, c: a flooding(70.5cm), b on land(52.7cm), c on land(32.3cm) | Transects, position a, b, c: a S. boganidensis (4) b S. boganidensis (4) c S. boganidensis (1), S. glauca (3) | 70.55 | 148.26 |
| 16SKB | 20160723 | K river | Transects, position a, b, c: a flooding, b flooding(60cm), c flooded(66.2cm) | Transects, position a, b, c: a S. glauca (4) b S. glauca (4) c S. glauca (4) | 70.55 | 148.16 |
| 16SBoydom | 20160723 | I river | Transects, position a, b, c: a flooding, b on land(52.7cm), c on land(42.5cm) | Transects, position a, b, c: a S. richardsonii (4) b S. richardsonii (4) c S. richardsonii (4) | 70.64 | 148.15 |
| 16SK | 20160723 | K river | Flooding and on land | S. richardsonii (6), S. pulchra (2) | 70.56 | 148.26 |
| 16SK4 | 20160723 | K river | Flooding and on land | S. pulchra (4), S. boganidensis (4) | 70.59 | 148.07 |
| 16SK5 | 20160723 | K river | Flooded and on land | S. richardsonii (3), S. pulchra (5) | 70.57 | 148.11 |
| 16SK7 | 20160723 | K river | Flooded and on land | S. boganidensis (8) | 70.54 | 148.23 |
| 16SAllaiha\* | 20160724 | I river | Flooding and on land | S. boganidensis (2), S. richardsonii (6), S. pulchra (3) | 70.52 | 147.30 |
| 16SAllaiha-hill\* | 20160724 | Hill | On hill | S. boganidensis (1), S. glauca (2), S. richardsonii (1) | 70.52 | 147.28 |
The Site No. in bold with asterisk is not shown in Figure 2.
