# Supplementary figures and images for: Effect of floods on the δ13C values in plant leaves: a study of willows in Northeastern Siberia
Source: PeerJ. 2018 Sep 20;6:e5374. doi: 10.7717/peerj.5374 (PMC6151259; doi:10.7717/peerj.5374)

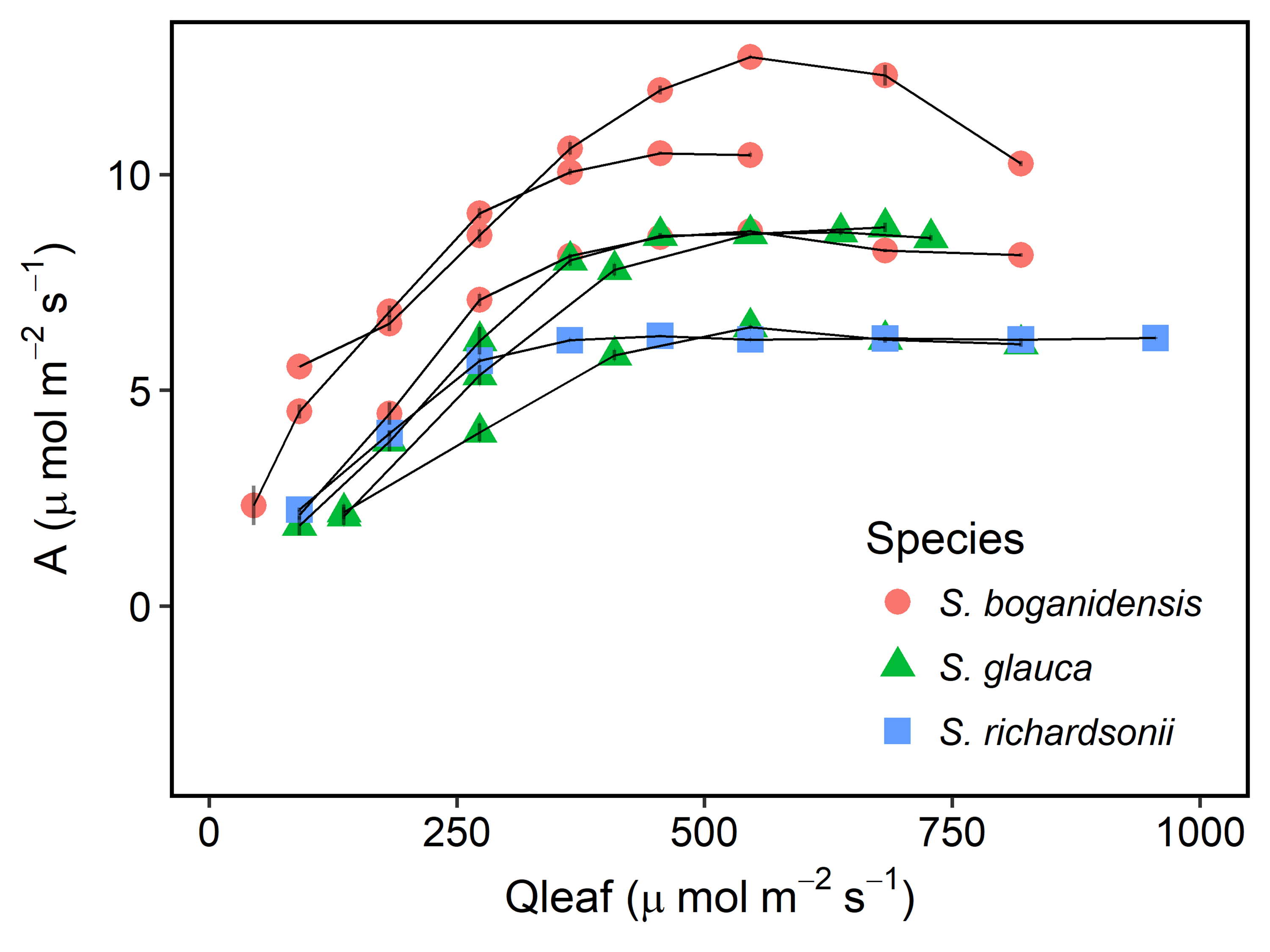

Supplement: Supplemental Information 3 — Photosynthesis light response curve of the willows S. boganidensis (red cycles), S. glauca (green triangles), and S. richardsonii (blue squares) under the light levels from 10 to 955 μmol m−2 s−1. The values were reported as means ± SD. [file peerj-06-5374-s003.png]

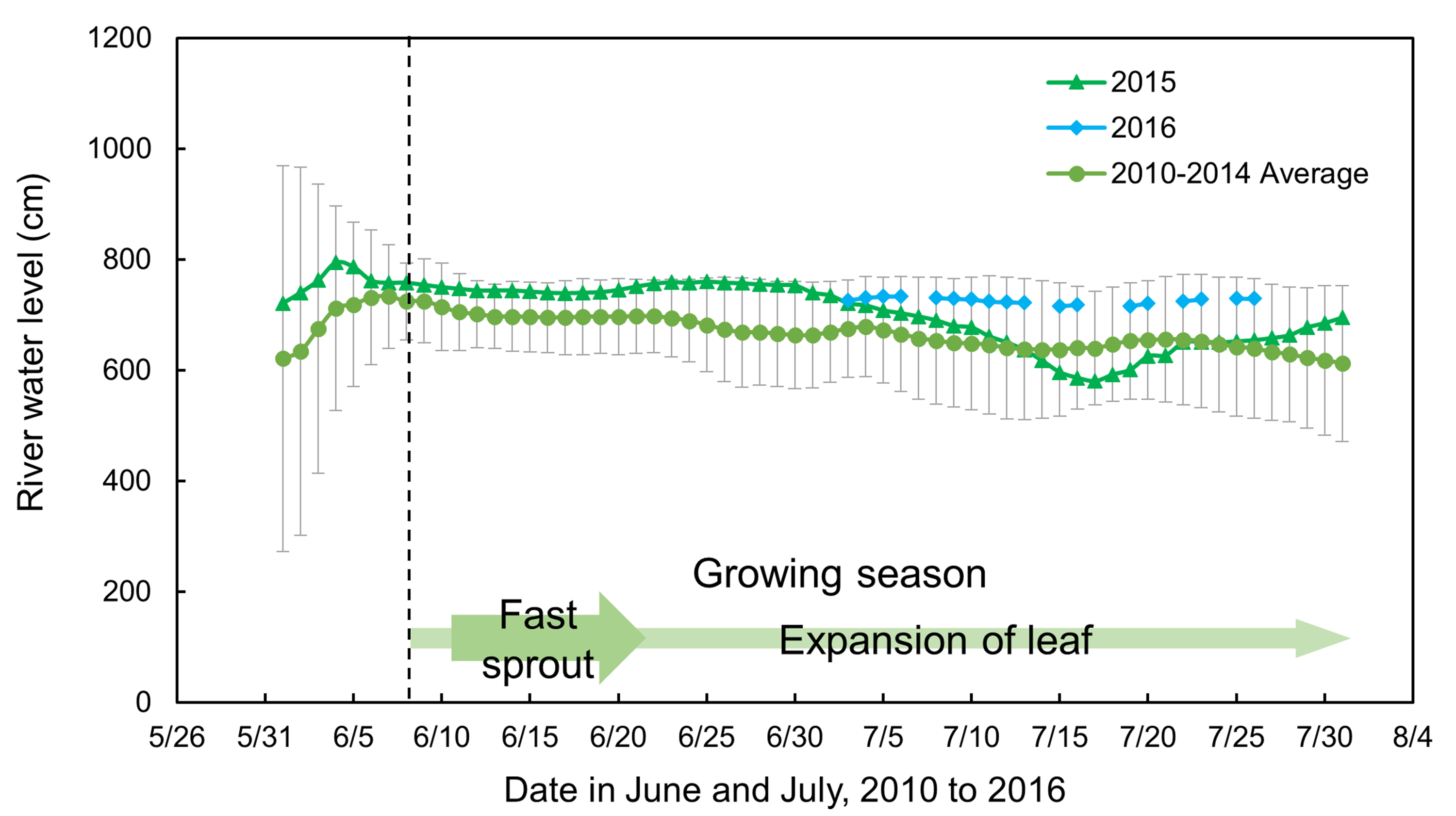

Supplement: Supplemental Information 4 — The average river water level during 2010-2014 was shown in circles, the water level in 2015 and 2016 was shown in triangles and diamonds, respectively. The arrow lines indicate the observed growing season, with fast growing period in wide arrow line and followed by slow growing period in narrow arrow line. [file peerj-06-5374-s004.png]

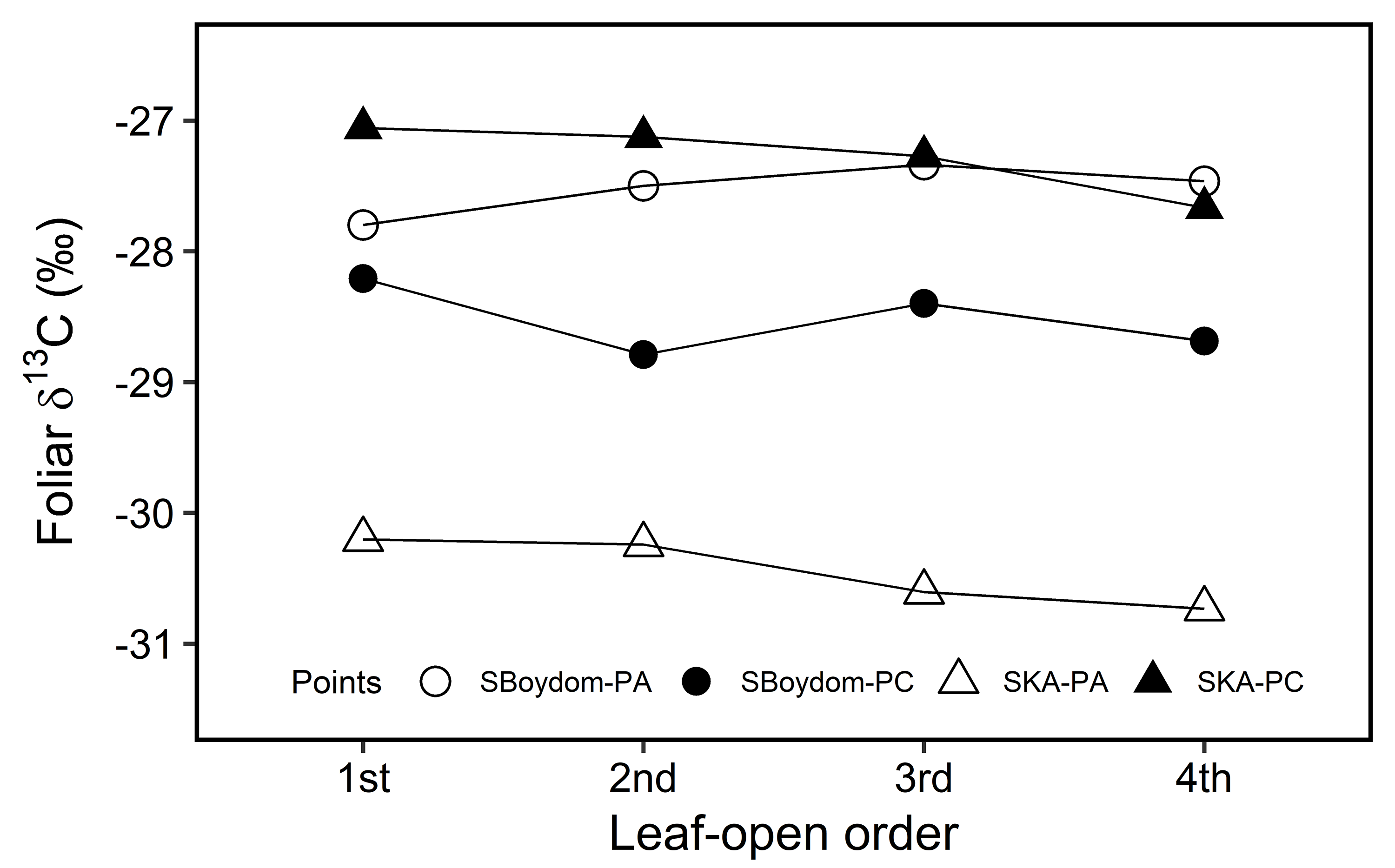

Supplement: Supplemental Information 5 — The foliar δ13C values (‰) of the leaves according to the leaf-open order, upon single shoot at each point of PA (empty symbols) and PC (filled symbols) in SBoydom (circles) and SKA (triangles). The different points are shown in different symbols. [file peerj-06-5374-s005.png]
